# The Inhibition of Mast Cell Activation of Radix *Paeoniae alba* Extraction Identified by TCRP Based and Conventional Cell Function Assay Systems

**DOI:** 10.1371/journal.pone.0155930

**Published:** 2016-05-19

**Authors:** Huiying Fu, Hongqiang Cheng, Gang Cao, Xingde Zhang, Jue Tu, Mingjiao Sun, Xiaozhou Mou, Qiyang Shou, Yuehai Ke

**Affiliations:** 1 Program in Molecular Cell Biology, Department of Basic Medical Sciences, Zhejiang University School of Medicine, Hangzhou, China; 2 Experimental Animal Research Center, Zhejiang Chinese Medical University, Hangzhou, China; 3 Central Laboratory, The Second Clinical Medical College, Zhejiang Chinese Medical University, Hangzhou, China; 4 Research Center of TCM Processing Technology, Zhejiang Chinese Medical University, Hangzhou, China; 5 School of Pharmacy, Nanjing Chinese Medical University, Nanjing, China; Macau University of Science and Technology, MACAO

## Abstract

Chinese herbs have long been used to treat allergic disease, but recently the development was greatly impeded by the lack of good methods to explore the mechanism of action. Here, we showed the effects of Chinese herb Radix *Paeoniae alba* were identified and characterized by a mast cell activation assay that involves electronic impedance readouts for dynamic monitoring of cellular responses to produce time-dependent cell responding profiles (TCRPs), and the anti-allergic activities were further confirmed with various conventional molecular and cell biology tools. We found Radix *P*. *alba* can dose-dependently inhibit TCPRs, and have anti-allergic function *in vitro and in vivo*. Radix *P*. *alba* suppressed mast cell degranulation not only inhibiting the translocation of granules to the plasma membrane, but also blocking membrane fusion and exocytosis; and that there may be other anti-allergic components in addition to paeoniflorin. Our results suggest that Radix *P*. *alba* regulated mast cell activation with multiple targets, and this approach is also suitable for discovering other mast cell degranulation-targeting Chinese herbs and their potential multi-target mechanisms.

## Introduction

Traditional Chinese Medicine (TCM) has been used as front-line pharmacotherapy for various diseases for many millennia in China. However, the development of TCM was greatly impeded owing to the lack of modern standards for the complexity of Chinese herb ingredient [1]. While no matter a single herb or herbal formula contains thousands of components that simultaneously modulate multiple pharmacological targets, which may be one of the main reasons for their observed therapeutic effects beyond the capabilities of a single compound, as well as the less adverse effects for disease prevention and chronic conditions compared to those of Western medicine [2–5]. Thus, methods for the assessment of the efficacy of TCM with multiple, interactive measures are required in order to promote its modernization.

To address this issue, several cell-based, high-throughput phenotypic approaches have been developed to determine the global responses of the targets of natural products to specific perturbations. The living cell morphological profiling method, can be used to dynamically monitor the cellular response to treatments by producing time-dependent cell response profiles (TCRPs)[6]. With this method, cells were seeded onto the surface of microelectronic cell sensor arrays integrated into the bottom of microtiter plates, and dynamically monitored cell-substrate impedance as reflected with Cell Index (CI)[7]. It is suggested the CI value basically correlates with three cellular parameters, namely cell number, morphology and attachment quality. All three parameters are intricately linked to signaling pathways regulating various facets of cellular physiology, and therefore this approach allows for expansion of the “biological space” at which a Chinese herb is screened and provides ample opportunity detect and identify biological activity associated with drugs in an unbiased manner [6, 8, 9].

Radix *Paeoniae alba* (Radix *P*. *alba*) is a Chinese herb, which has been documented to treat allergic disorders in traditional medical clinics [10, 11]. The extraction of total glucosides from peony was approved as an anti-inflammatory drug by the China Food and Drug Administration. However, no data are available on the mechanism of action of the anti-allergic effect of Radix *P*. *alba*. We therefore set out to analyze the anti-allergic effect of Radix *P*. *alba* with TCRP technology and determine the potential mechanisms.

## Materials and Methods

### Cell Culture

Rat basophilic leukemia (RBL-2H3) cells (American Type Culture Collection; Manassas, VA) were cultured in a humidified incubator at 37°C and 5% CO_2_ with Dulbecco’s modified Eagle's medium (DMEM) containing 10% fetal bovine serum.

### Reagents and Antibodies

All the reagents were purchased from Sigma-Aldrich (St. Louis, MO) unless indicated otherwise. Phorbol 12-myristate 13-acetate (PMA) was obtained from Beyotime Institute of Biotechnology (Jiangsu, China). Adenosine was obtained from the National Institutes Food and Drug Control, China (NIFDC). *Gab2* short interfering RNA (siRNA) was purchased from Santa Cruz Biotechnology (Santa Cruz, CA). The Cyto Tox96 Non-Radioactive Cytotoxicity assay kit was obtained from Promega (Madison, WI).

### Extraction and Separation

Radix *P*. *alba* (see S1 Fig) was cleaned, and crushed, and added with distilled water to ultrasonic extraction and decoction treatments for 30 min each. After centrifugation at 10000 rpm for 15 min, the supernatant was used for screening with the xCELLignece system (ACEA, CA).

To isolate the various ingredients of Radix *P*. *alba*, a dried sample (6 g) was pulverized and extracted three times with boiled water (80:1, v/w). The water solution was further extracted with petroleum ether (1:1, v/v). The petroleum ether extract was evaporated to dryness to obtain the petroleum ether component (PE). The water solution was sequentially extracted with ethyl acetate, n-butyl alcohol, and ethanol (1:1, v/v each), and the extracts were evaporated to dryness to obtain the ethyl acetate (EA), n-butyl alcohol (NBA), and ethanol (EtOH) components, respectively. The ethanol precipitate was evaporated to obtain the H_2_O components.

### TCRPs Detected Using the xCELLigence System

TCRPs were recorded with the xCELLigence system as described previously [6, 12, 13], with 3 independent experiments run in triplicate for each test. Briefly, 50 μL of complete DMEM was added to 96-well E-Plates to obtain background readings before the addition of 100 μL of the cell suspension. After incubating at room temperature for 30 min, the E-Plates were placed onto the RTCA SP station in the incubator and the CI was recorded. The cells were allowed to grow for 18–24 h before the addition of the indicated extracts of Radix *P*. *alba* or compounds. The cells were monitored every 2 min for more than 3 h after treatment in order to capture the short-term TCRPs.

### Cell Morphology Analysis Based on an Immunofluorescence Assay

RBL-2H3 cells were seeded in 24-well tissue culture plates containing glass coverslips and allowed to attach and spread for 24 h. The cells were treated with various antigens, including dinitrophenyl-bovine serum albumin (DNP-BSA; Invitrogen Molecular Probes; Carlsbad, CA), adenosine, and PMA, for the indicated times, and then fixed with 4% paraformaldehyde for 15 min. The cells were permeabilized in phosphate-buffered saline (PBS) containing 0.5% Triton X-100 for 15 min and blocked with PBS containing 1% BSA for 1 h. The following primary and secondary stainings were performed: CD63 antibody (Epitomics, Inc.; Burlingame, CA) at a dilution of 1:50, fluorescein isothiocyanate (FITC)-conjugated anti-rabbit IgG (MultiSciences Biotech Co.; Hangzhou, China) at 1:100, FITC-conjugated tubulin antibody (Cell Signaling Technology; Beverly, MA), phalloidin-rhodamine (Invitrogen Molecular Probes; Carlsbad, CA), and 4′6-diamidino-2-phenylindole (DAPI; Invitrogen). Fluorescence was visualized and imaged with Laser Scanning Microscope 510 META (Carl Zeiss; NY, USA).

### High-Performance Liquid Chromatography (HPLC)

HPLC analyses of Radix *P*. *alba* and its extracts were performed with the Agilent XDB-ODS column (250 × 4.6 mm, 5 μm diameter). The mobile phase was acetonitrile (A) and 0.1% phosphoric acid (B). The following gradient elution mode was applied: 0–8 min, 5–9% A; 8–20 min, 9–15% A; 20–25 min, 15–17% A; 25–40 min, 17% A; 40–45 min, 17–30% A; 45–60 min, 30% A. The detection wavelength was 230 nm, the flow rate was 1 mL/min, and the column temperature was 25°C.

### Degranulation Assay

RBL-2H3 cells were cultured in 24-well plates for 24 h, washed, incubated in Tyrode buffer, and then stimulated with the following antigen: 100 ng/mL DNP-BSA (after incubation with 100 ng/mL anti-DNP-IgE for 12 h), PMA, or adenosine for 30 min. Degranulation was determined based on β-hexosaminidase release as described previously [14].

### Animal Model and Administration

Adult male ICR mice (SLRC Laboratory Animals, Shanghai, China) weighing 18–22 g were used in the experiments. The protocol was approved by the Committee on the Ethics of Animal Experiments of Zhejiang Chinese Medical University. All surgery was performed under sodium pentobarbital anesthesia, and all efforts were made to minimize suffering. Radix *P*. *alba* at various concentrations (800 mg/kg, 400 mg/kg, 200 mg/kg) or dexamethasone (1.0mg/kg) was administered intragastrically for 6 days following sensitization with dinitrofluorobenzene (DNFB) (Shanghai Chemical Reagent Company, Shanghai, China) onto the shaved abdominal skin of the mice. The negative control mice were normally sensitized and painted with olive oil alone when challenged. The positive control mice with contact dermatitis were given normal saline. Indexes, including immunohistochemistry of the ear with hematoxylin and eosin staining (original magnification ×200), and ear weights were assayed after the second challenge on the ears with DNFB[15, 16].

### Calcium Measurements

Calcium levels were assayed using the Fluo4-AM Calcium Indicator (Dojindo; Kumamoto, Japan). The cells were grown in a 96-well plate for 24 h, the various extractions of Radix *P*. *alba* were added for 2 h, and then the plates were washed with Hanks’ balanced salt solution (HBSS) three times. The cells were loaded with Fluo4-AM dye for 30 min at 37°C and washed with HBSS three more times, followed by the addition of stimulants. The changes in dye fluorescence were monitored on the SpectraMax M5 Multifunctional microplate reader (Molecular Devices; Sunnyvale, CA). [Ca^2+^]_i_ mobilization was represented as expressed as the measured of fluorescence intensity value.

### Immunoblotting

IgE-sensitized RBL-2H3 cells were pretreated with Radix *P*. *alba* and its fractions for 2 h and then stimulated with 100 ng/mL DNP-BSA for 30 min. Equal amounts of cellular protein were resolved by 10% sodium dodecyl sulfate-polyacrylamide gel electrophoresis and transferred onto nitrocellulose membranes. After blocking, the membranes were incubated with the primary antibodies, p-Erk/Erk and p-Akt/Akt (Cell Signaling Technology), and IRDye800 goat anti-Rabbit IgG secondary antibody (Li-Cor; Lincoln, NE). Immunoreactive proteins were visualized using the Odyssey Infrared Imaging System (Li-Cor).

### Statistical Analysis

All data are reported as the means ± S.E.M. Data were analyzed using standard statistical methods of one-way ANOVA followed by Student’s t-tests using SPSS 15.0 software. P < 0.05 was considered as statistically significant.

## Results

### Recording Mast Cell Degranulation with TCRPs

**Fig 1:**
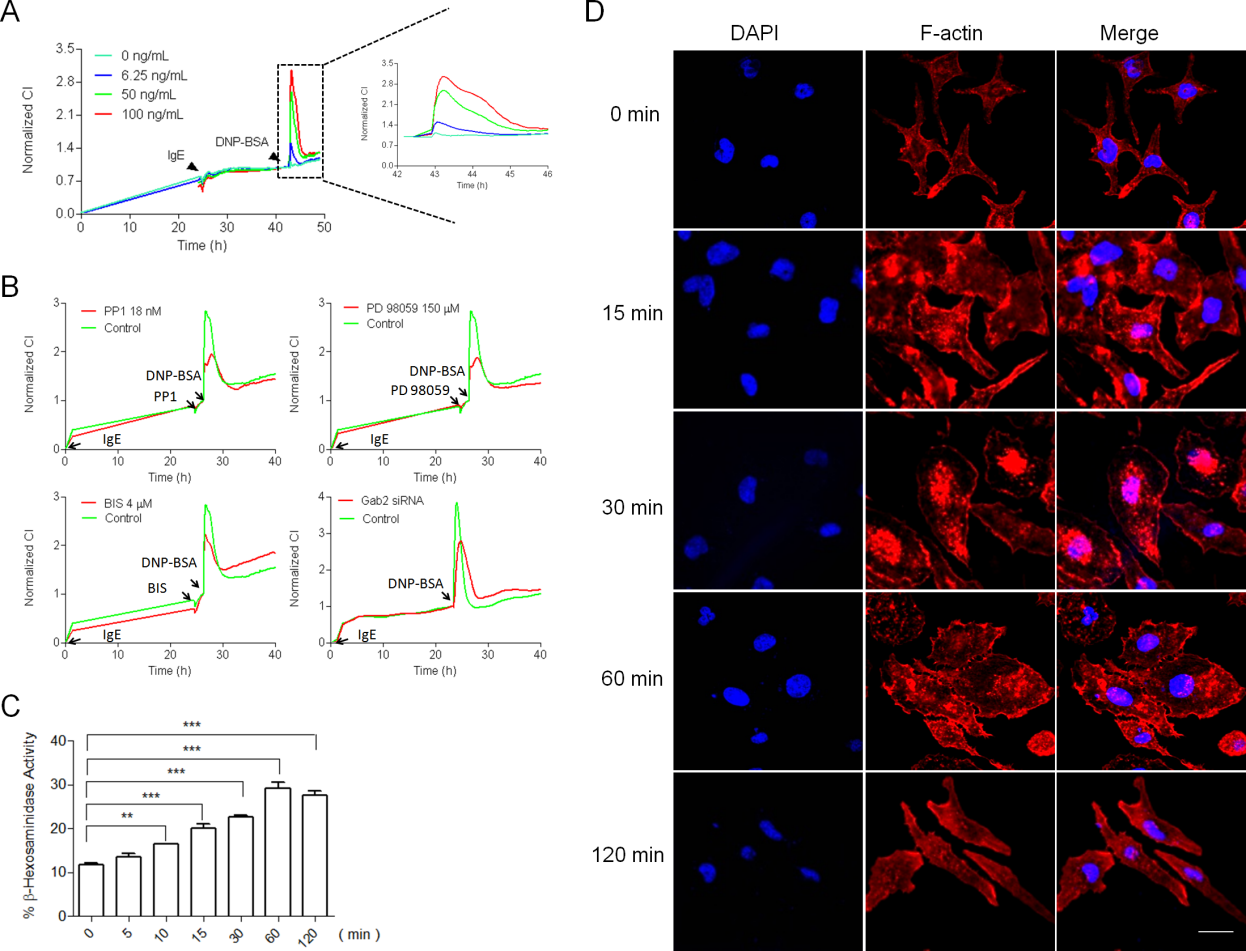
Construction of IgE-mediated mast cell TCRPs. (A) IgE-mediated TCRPs for functional monitoring of mast cell degranulation. RBL-2H3 mast cells were cultured on E-plates at a density of 10,000 cells/well, and incubated with 100 ng/mL anti-DNP IgE approximately 22 h later. After sensitization overnight, the cells were stimulated with 100 ng/mL DNP-BSA. The impedance expressed as the cell index (CI), was continuously monitored. (B) Pharmacological inhibitors of FcεRI receptor-activated downstream signal pathways that significantly attenuated IgE-mediated TCRPs. IgE sensitized RBL-2H3 cells were seed on the E-plates overnight at a density of 20,000 cells/well, and pre-treated with the inhibitors PP1, PD98059, bisindoylmaleimide I and *Gab2* siRNA, before stimulation with DNP-BSA. (C) Mediator release assay of RBL-2H3 mast cell activation. RBL-2H3 cells were sensitized with 100 ng/mL IgE and activated by the addition of 100 ng/mL DNP-BSA. β-hexosaminidase activities were measured at the indicated time. **, P < 0.01 vs. 0 min. Bar 10 μm. (D) RBL-2H3 mast cell activation was accompanied by morphological changes. The cells were sensitized with IgE and stimulated with DNP-BSA, and were then fixed with paraformaldehyde at the indicated time points. F-actin was stained with rhodamine-pholloidin as described in the Methods.

In mast cells, crosslinking high-affinity IgE receptor (FcεRI) stimulation with an antigen results in the degranulation of secretory vesicles accompanied by dramatic remodeling of the cellular cytoskeleton [17–19], and this process can be recorded and characterized using TCRPs. Representative dose-dependent TCRPs produced by IgE-mediated RBL-2H3 cell activation were shown in Fig 1A, which displayed the impedance between the cell memberane and the sensor surface correlating with cell morphology and adhesion. Activation of the FcεRI receptor results in subsequent activation of downstream signaling pathways involving phosphorylation and dephosphorylation of a number of signal proteins that are indispensable for mast cell degranulation. Therefore, we selected some reported pharmacological inhibitors to pretreat IgE-sensitized RBL-2H3 cells, which were then stimulated with DNP-BSA. As shown in Fig 1B, the TCRPs revealed that certain protein kinase inhibitors impaired IgE-mediated mast cell degranulation, which is in agreement with previous reports [20–22]. The *Gab2* mRNA expression level and mast cell degranulation was decreased by *Gab2* siRNA treatment (see S2 Fig). To determine whether the IgE-mediated TCRP was associated with RBL-2H3 mast cell activation, IgE-mediated morphological changes and mediators release were measured. As seen in Fig 1C and 1D, aggregation of the IgE-bound FcεRI receptor triggered the release of β-hexosaminidase, a marker for mast cell degranulation, as well as time-dependent dynamic reorganization of the actin cytoskeleton. Taken together, these results show that impedance values are in line with the change of morphological dynamics and mediator release, and the IgE-mediated impedance-based mast cell TCRP could fully reflect the mast cell degranulation process. Therefore, this approach is suitable for high-throughput screening of compounds or Chinese herbs that target mast cell degranulation.

### Mast Cell TCRP-Directed Screening of Radix *P*. *alba*

**Fig 2:**
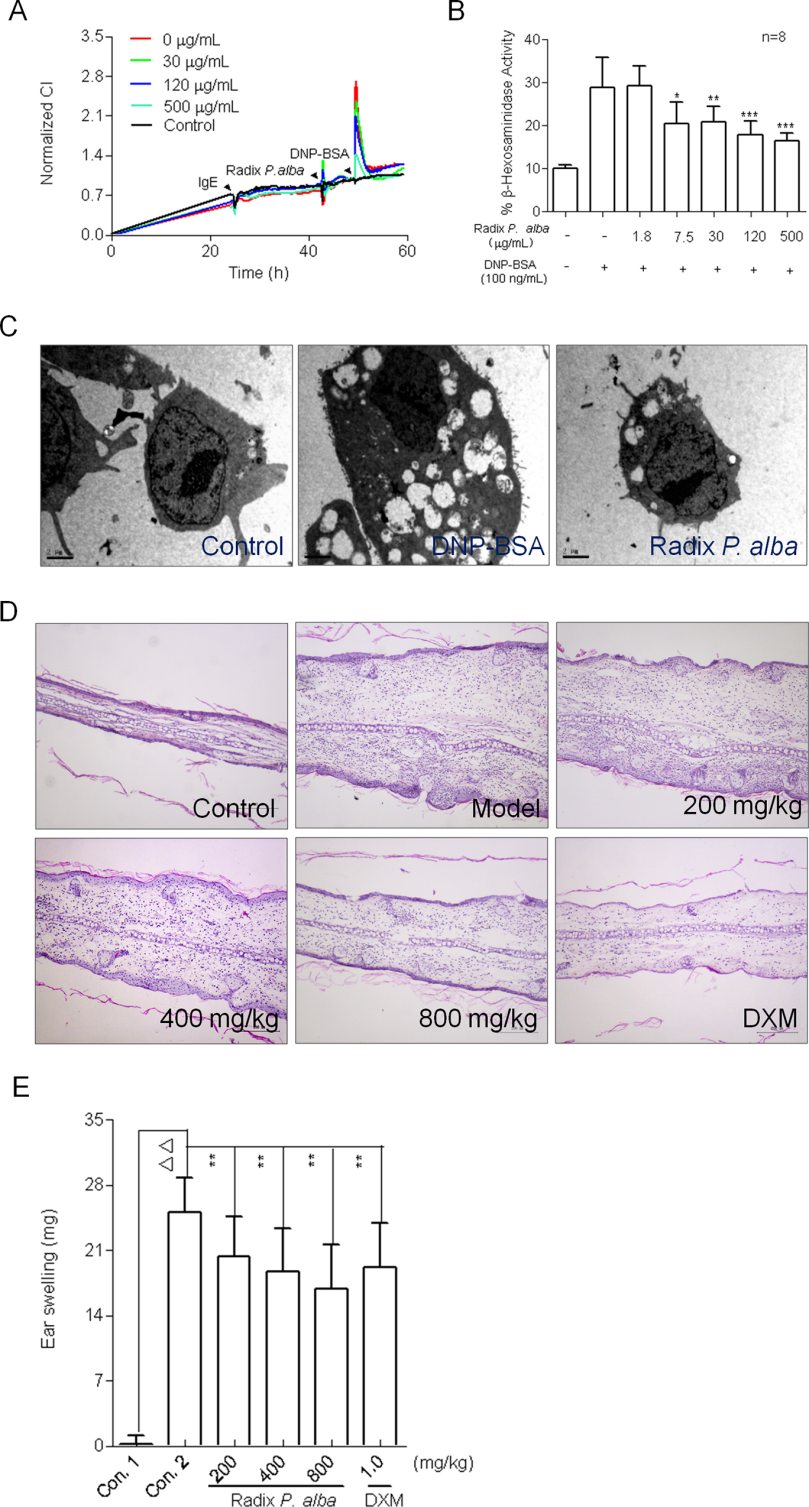
Validation of the anti-allergic effect of Radix *P*. *alba in vitro* and *in vivo*. (A) Typical dynamic cell response curves are indicative and predictive for inhibition of mast cell activation. IgE-sensitized RBL-2H3 cells were incubated with different concentrations of Radix *P*. *alba* 1 h prior to the addition of 100 ng/mL DNP-BSA. The cell index (CI) values were continuously assayed for over 3 h. (B) Effect of Radix *P*. *alba* on mast cell degranulation. The cells sensitized with 100 ng/mL IgE were incubated with the different concentrations of Radix *P*. *alba* and subsequently actived by 100 ng/mL DNP-BSA. β-hexosaminidase activity was measured as described in Methods. (C) TEM images of the vehicle fusion in mast cells treated with or without Radix *P*. *alba*. The cells were sensitized with 100 ng/mL IgE overnight and pre-treated with 500 μg/mL Radix *P*. *alba* before stimulation with DNP-BSA. After stimulation for 30 min, the cells were fixed with 2.5% glutaraldehyde, and then stained, dehydrated, and embedded. The ultrathin sections were observed using a Tecnai 10 transmission electron microscope. (D) Hematoxylin and eosin-stained sections of the ears prepared 24 h after the challenge. (E) Effect of Radix *P*. *alba* on the DNFB-induced ear swelling response in mice. It was determined by ear punch weight determinations. Values are means ± SEM of 12 mice; * P < 0.05, ** P < 0.01 vs. positive control, △△ P < 0.01 vs. negative control.

Then we use this mast cell TCPR to identify the effects of Radix *P*. *alba*. As shown in Fig 2A, Radix *P*. *alba* dose-dependently attenuated the impedance response of the IgE-mediated TCRP under the non-cytotoxic dose (S3 Fig). To verify the hypothesis that a decrease of the CI in TCPR is predictive of inhibiting mast cell activation. The activity of β-hexosaminidase released by activated mast cells was further determined. The results showed Radix *P*. *alba* significantly reduced the release of β-hexosaminidase (Fig 2B), and inhibited vesicle fusion in activated mast cells (Fig 2C).

Furthermore, we evaluated the anti-allergic activity of Radix *P*. *alba in vivo* using a DNFB induced ear swelling mouse model. Ear swelling was induced by placing DNFB into the ear and was assessed by measuring ear weight 24 h later. The inflamed auricles of the mice were examined histologically following hematoxylin and eosin staining. Severe inflammation such as edema, epidermal hyperplasia, and infiltration of inflammatory cells was observed in DNFB-treated mouse ears (Fig 2D). As expected, Radix *P*. *alba* treatment ameliorated inflammation of the epidermis of mice just as well as treatment with the positive control drug dexamethasone (Fig 2D). Application of DNFB resulted in a significant increase in ear weight, which was greatly reduced by Radix *P*. *alba* treatment (Fig 2E).

### Microtubule-Dependent Granule Translocation and Calcium-Dependent Membrane Fusion and Exocytosis Based on TCRP

**Fig 3:**
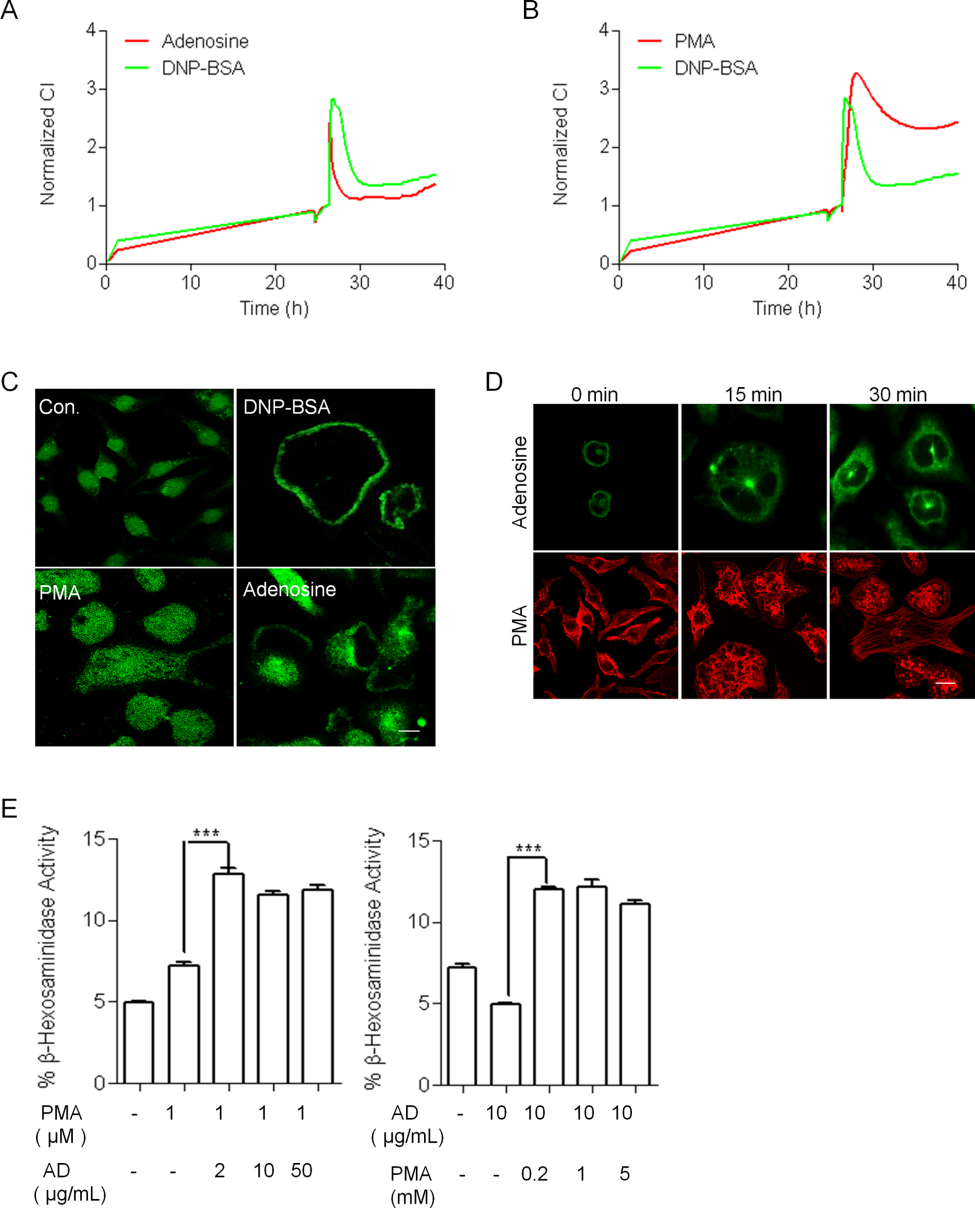
Adenosine and PMA-induced mast cell TCRPs. (A-B) IgE-mediated TCRP comparison with cell-electrode impedance dynamics induced by adenosine or PMA. RBL-2H3 cells were seeded on the E-plates overnight at a density of 20,000 cells/well, and were then treated with DNP-BSA (IgE-sensitized) and adenosine or PMA (no sensitization). (C) Representative distribution of CD63 stimulated by different antigens in RBL-2H3. RBL-2H3 cells with or without IgE sensitization were stimulated with DNP-BSA, adenosine, and PMA for 30 min. Bar, 5 μm. (D) Adenosine induced microtubule formation whereas PMA induced F-actin disassembly. Treated cells were fixed and processed for staining with phalloidin-rhodamine (red) and antibody to β-tubulin (green). Bar, 5 μm. (E) Adenosine together with PMA induced β-hexosaminidase release. The cells were seeded in 24-well plates overnight, pretreated with PMA or adenosine and subsequently activated with different concentrations of adenosine or PMA. *** P < 0.001 vs. with PMA or adenosine only.

Recent studies have demonstrated that FcεRI-dependent degranulation involves at least two processes: (1) the calcium-independent, microtubule-dependent translocation of granules to the plasma membrane and (2) calcium-dependent membrane fusion and exocytosis [18, 23]. The two main events of mast cell degranulation involve different signaling cascades. The microtubule-dependent translocation of granules to the plasma membrane depends on the phosphoinositide 3 kinase (PI3K)/AKT signaling pathway, whereas calcium-dependent membrane fusion and exocytosis require protein kinase C (PKC) signaling [18]. We examined TCRPs treated with adenosine (Binding to A3R GPCRs and leading to the activation of PI3Kγ)[24] and PMA (a PKC agonist). As shown in Fig 3A and 3B, both adenosine and PMA caused an immediate and transient increase with a subsequent decrease in the CI, but there were differences in the duration time to complete the response. Therefore, each treatment showed its own characteristic ‘signature’ pattern. To determine whether adenosine and PMA stimulated mast cell degranulation, we examined the staining distribution pattern of CD63, a broadly expressed transmembrane protein [25], and determined the level of β-hexosaminidase release. After stimulation with DNP-BSA or adenosine, the granules labeled with CD63 were translocated to the plasma membrane in RBL-2H3 cells, but this did not occur in cells treated with PMA (Fig 3C). Furthermore, neither adenosine nor PMA induced β-hexosaminidase release, which agrees with previous reports by Yanase et al. [19] and Nunomura et al. [26] (S4A Fig). In addition, we observed enhancement of the formation of microtubule in cells treated with adenosine, and membrane ruffling of the cortical F-actin in cells treated with PMA, as shown in Fig 3D. Moreover, adenosine amplified the effects of DNP-BSA-stimulated mast cell degranulation [26]; however, this result was not observed in PMA-treated cells (S4B Fig). Because vesicle translocation and PKC-dependent Ca^2+^ activation are two of the most important processes for mast cell degranulation, we examined whether RBL-2H3 cells stimulated with both adenosine and PMA showed induced degranulation. Indeed, regardless of the order of pre-treatment or concentration of adenosine and PMA, the combined treatment induced mast cell degranulation in all cells (Fig 3E). These results indicated that the adenosine and PMA TCRPs could respectively record mast cell-activated processes based on the translocation of vesicles and exocytosis-mediated cytoskeleton rearrangement.

### Characterization of Multi-Targets in the Chinese Herb Radix *P*. *alba*

**Fig 4:**
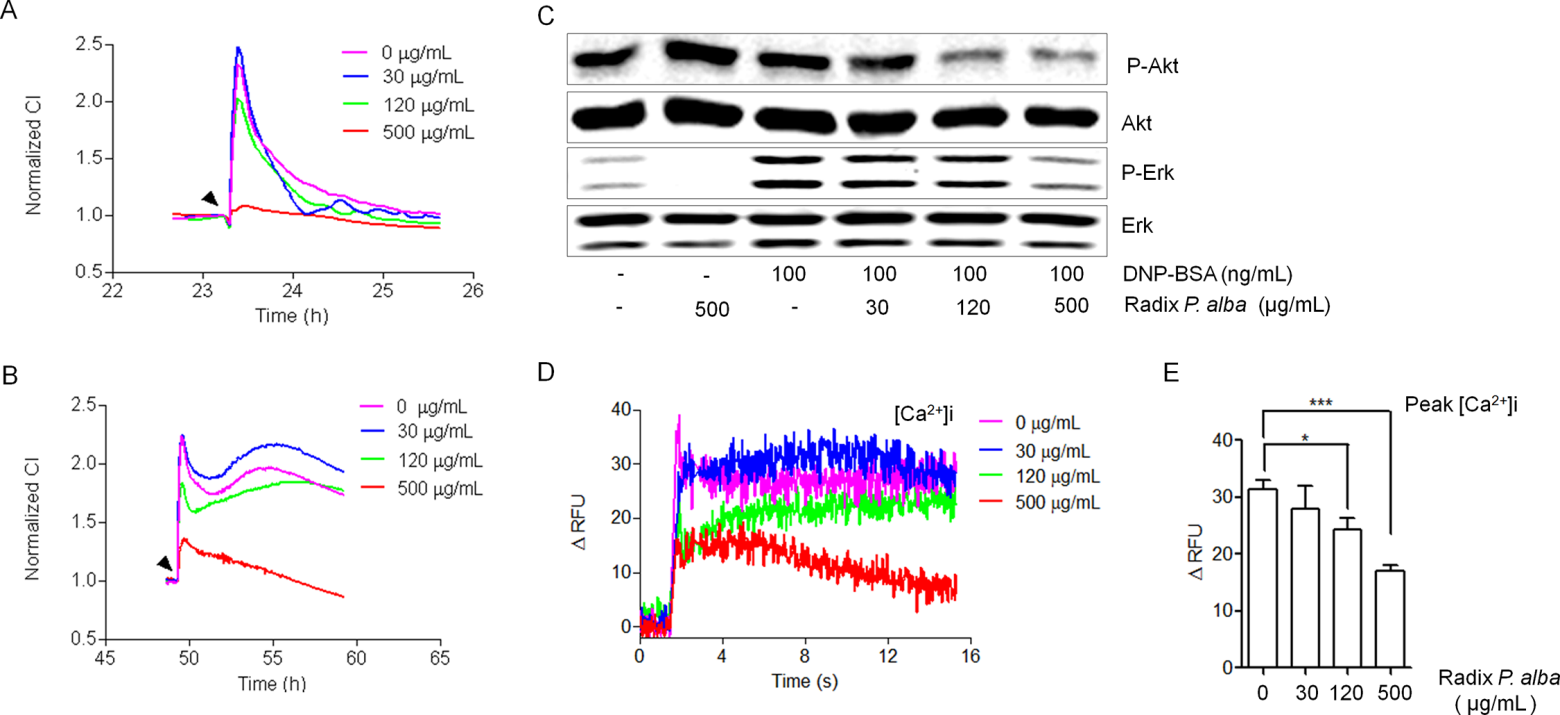
Mast cell TCRPs-based screening and identification of multi-targets of Radix *P*. *alba*. (A) Adenosine-mediated TCRP-based functional screening of Radix *P*. *alba*. RBL-2H3 cells were seeded on the E-plates overnight and pretreated with various concentrations of Radix *P*. *alba* for 1 h before the addition of 10 μg/mL adenosine. (B) PMA TCRP-based functional screening of Radix *P*. *alba*. RBL-2H3 cells were seeded on E-plates overnight and pretreated with various concentrations of Radix *P*. *alba*, as indicated, for 1 h before the addition of 1 μm PMA. (C) Immunoblotting analysis of the phosphorylation of Akt and Erk1/2 in RBL-2H3 cells. IgE-sensitized RBL-2H3 cells were pre-treated with Radix *P*. *alba* for 1 h and with 100 ng/mL DNP-BSA for 30 min. The cells were then lysed and immunoblotted with anti-p-Akt, anti-Erk1/2, and anti-p-Erk1/2 antibodies. (D-E) Effects of Radix *P*. *alba* on [Ca^2+^]_i_ mobilization. RBL-2H3 cells were treated with Radix *P*. *alba* for 1 h and with 50 μL of 5 mM Fluo-4/AM for 30 min at 37°C. The cells were monitored in real-time by spectra Max M5 after stimulation with PKC. The [Ca^2+^]i mobilization is expressed as the relative fluorescence intensity and the values were normalized to the control values. * P < 0.05, *** P < 0.01 vs. control.

The effectiveness of TCM for the treatment and prevention of various diseases has been well documented, and tends to show a multi-target pattern in its mechanism. Indeed, the underlying theory of TCM is rooted in the fundamental principle that Chinese herbs regulate and restore the balance of a disorder of the human body through multiple targets and components [3, 27]. We next investigated whether Radix *P*. *alba* could suppress mast cell degranulation in a multi-targeted manner using various mast cell activation TCRPs. First, we tested the effect of Radix *P*. *alba* on the adenosine and PMA TCRPs. As shown in Fig 4A and 4B, Radix *P*. *alba* inhibited the mast cell activation that was stimulated by either adenosine or PMA in a dose-dependent manner. To determine the specific activity of Radix *P*. *alba*, we further detected the changes of phosphorylated Akt and Erk (p-Akt and p-Erk, respectively) levels in mast cell degranulation. The results showed a dose-dependent decrease of p-Akt and p-Erk levels in Radix *P*. *alba*-treated cells (Fig 4C). Measurement of Ca^2+^ influx also showed that stimulation of IgE-sensitized mast cells with DNP-BSA induced a rapid Ca^2+^ flux, which was dose-dependently inhibited by treatment of Radix *P*. *alba* (Fig 4D and 4E). These results demonstrated that Radix *P*. *alba* inhibits mast cell degranulation with a multi-target pattern, and that the application of TCRP technology represents a rapid analytical tool for elucidating the potential cellular mechanisms of Chinese herbs.

### TCRP-Directed Isolation of Radix *P*. *alba*

**Fig 5:**
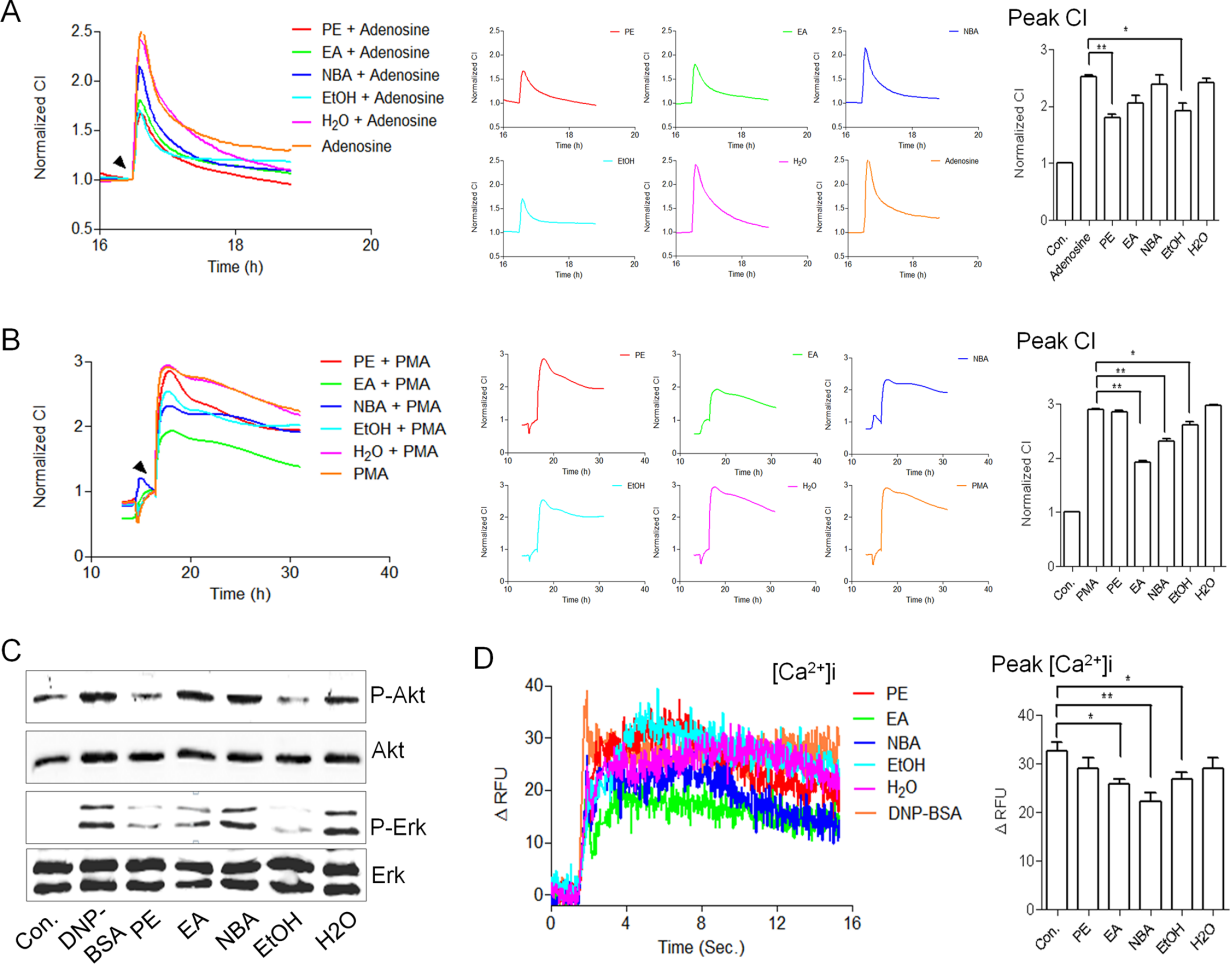
Mast cell TCRP-based identification of the bioactive ingredients of Radix *P*. *alba*. (A-B) Adenosine-mediated TCRP (A) and PMA-mediated TCRP (B) for functional screening of various Radix *P*. *alba* components. For details about the five various components, see the Materials and Methods. (C) Immunoblotting analysis of the phosphorylation of Akt and Erk1/2 in RBL-2H3 cells treated with different Radix *P*. *alba* components. (D) Effects of the five fractions on [Ca^2+^]_i_ mobilization. RBL-2H3 cells were treated with PE, EA, EtOH, and H_2_O extractions, respectively, for 1 h and incubated with 50 μL of 5 mM Fluo-4/AM for 30 min at 37°C after washing three times with HBSS. The cells were monitored in real-time by spectra Max M5 pre- and post-stimulation with PKC. The [Ca^2+^]_i_ mobilization was statistically analyzed by comparing the peak fluorescence intensity value which were normalized to the control values. * P < 0.05, ** P < 0.01 vs. control.

To investigate the active ingredients inhibiting the mast cell activation in Radix *P*. *alba*, we introduced mast cell TCRPs for identifying active components in combination with chemical separation techniques. The Radix *P*. *alba* crude exact was serially extracted with organic solvents and separated into five fractions. The fractions extracted with PE and EtOH dramatically decreased the peak values of adenosine-mediated TCRP (Fig 5A), which correspondingly down-regulated the p-Akt and p-Erk levels (Fig 5C). The EA, NBA, and EtOH fractions significantly reduced the peak values of the PMA-mediated TCRP (Fig 5B). To further verify these effects, we analyzed the Ca^2+^ influx of RBL-2H3 cells triggered by DNP-BSA in the presence or absence of the different fractions. The EA, NBA, and EtOH fractions significantly decreased the Ca^2+^ influx (Fig 5D), which was consistent with the TCRPs. We identified the components of the five fractions with HPLC analysis, and found that the PE fraction did not contain paeoniflorin, a known anti-allergic ingredient of Radix *P*. *alba* [28–31] (S5A Fig). This suggested there may be other active components present. We further examined the effects of the different fractions on β-hexosaminidase release. The PE, EA, and NBA fractions all significantly decreased the β-hexosaminidase release levels, while the EtOH fraction only slightly inhibited the degranulation level (S5B Fig). This may due to the relative differences in the inhibiting power of the different signaling pathways involved. Together, these results demonstrate the multi-targeted function of Radix *P*. *alba* produced by its various components (Table 1).

**Table 1:** Summary of the effects of five fractions.

	AD TCRPs	PMA TCRPs	Ca2+ influx	β-hexosaminidase release
PE	++	-	-	++
EA	-	++	+	+
NBA	-	++	++	++
EtOH	+	+	+	- (+)
H_2_O	-	-	-	-

“-”, Negative; “+”, Positive.

## Discussion and Conclusion

In this work, we used mast cell TCRPs to screen anti-allergic action of Chinese herb Radix *P*. *alba*. There are several important features that are critical for their application to natural product screening. First, the TCRP technology has the capacity to quantitatively measure minute changes in cell morphology with a very specific manner depending on the Rho family GTPase being activated. As we have known, IgE-mediated mast cell degranulation usually accompanies the actin cytoskeleton and cell morphology changes. While the actin cytoskeleton is that of a dynamic and plastic system that controls the intracellular functional signaling and not simply a static framework assigned to maintain cellular architecture. Therefore, the profiles generated with IgE-mediated mast cell degranulation are amenable to modulation by biologically active natural products in a manner that is dependent on mechanism and concentration of the sample being tested. Radix *P*. *alba* initially screened by TCRP was suggested that it inhibited mast cell activation. We confirmed this hypothesis by *in vitro* and *in vivo* experiments, and the results showed Radix *P*. *alba* decreased allergic response by inhibiting mast cell degranulation.

The mast cell degranulation process can be dissected into two main events: the microtubule-dependent translocation of granules to the plasma membrane, and actin-dependent granule–plasma membrane fusion and exocytosis; and these two processes respectively depend on Gab2/PI3K/AKT signaling and calcium signaling, they allow for a second feature, which is that IgE-mediated TCRP can also be decomposed into two components: (1) granule-translocation TCRP stimulated by adenosine and (2) granule–plasma membrane fusion and exocytosis TCRP induced by PMA. Our results showed individually activating any of them couldn’t induce mast cell degranulation, but simultaneously could, which suggested these two pathways are essential for mast cell activation. We also found Radix *P*. *alba* not only inhibited adenosine TCRPs but also attenuated PMA TCRPs, which indicated Radix *P*. *alba* regulated mast cell degranulation by inhibiting the translocation of granules to the plasma membrane as well as membrane fusion and exocytosis, as further was confirmed with well-orchestrated traditional tools.

Radix *P*. *alba* as traditional medicine has the role of anti-pyretic and anti-inflammatory activity. Recent years, studies showed it and its formulas have anti-allergic effects, inhibiting ovalbumin-induced allergic rhinitis[10], IgE-mediated type I hypersensitivity response[32], and decreasing histamine release from rat mast cells. Bomi et al. found the main constituents paeonol and paeoniflorin both can inhibit the mast cell degranulation, while paeonol was more powerful one[32]. Myungsuk et al. detected the anti-allergic effects of seventeen isolated compounds from *Paeonia anomala* used in Mongolian traditional medicine, and found 7 compounds can inhibit mast cell degranulation[33]. These studies demonstrated there are multicomponent involved in inhibiting mast cell activation, as is also consistent with our results.

Together, Radix *P*. *alba* inhibited mast cell activation with multi-target, and this approach is also suitable for discovering other mast cell degranulation-targeting Chinese herbs and their potential multi-target mechanisms.

## Supporting Information

S1 Data Raw data. (RAR)

S1 Fig The HPLC analysis of Radix *P*. *alba*. The 0.5g powered medicinal materials was added 20 mL methanol, soaked for 4h, and ultrasonic treatment for 30 min. The filtrate was 0.45 μm for filtration. Elution condition sees the Methods.
(TIF)

S2 Fig Decreasing the degranulation of RBL-2H3 cells transfected with *Gab2* siRNA. (A) *Gab2* mRNA level assay of mast cells transfected with *Gab2* siRNA. (B) The β-hexosaminidase release of mast cells transfected with *Gab2* siRNA was decreased comparing with the only Lip 2000 treatment. The methods of β-hexosaminidase assay see the Methods.
(TIF)

S3 Fig The cytotoxicity examination of Radix *P*. *alba*. (A) The cytotoxicity observation of Radix *P*. *alba* with TCRP approach. RBL-2H3 cells were seed on the E-plate allowing to grow for 24 h, and then were added the various concentrations of Radix *P*. *alba* monitoring every 15 min for over 20 h. (B) Cytotoxicity assay of Radix *P*. *alba* on RBL-2H3 cells. RBL-2H3 cells were cultured in a 96-well plate at 10,000 cells per well for 24h and treated with various concentrations of Radix *P*. *alba* for 2h. The cell supernatant was collected and assayed the LDH release with the Cyto Tox96 Non-Radioactive Cytotoxicity assay kit following the manufacturer’s suggested protocol.
(TIF)

S4 Fig The effects of adenosine and PMA on mast cell degranulation response. (A) The β-hexosaminidase release assays of PMA or adenosine on mast cell alone. (B) The enhancing effects of adenosine on β-hexosaminidase release from mast cells sensitized with anti-DNP IgE, when the dose of DNP-BSA fail to induce degranulation. RBL-2H3 cells were sensitized with 100 ng/mL of IgE for 24 h and stimulated with adenosine or PMA for 1min, then added the DNP-BSA with the indicated concentrations for 30min.
(TIF)

S5 Fig The HPLC analyses and activity determination of the five fractions of Radix *P*. *alba*. (A) The HPLC profiles of the five fractions and the control substance Paeoniflorin. (B) The inhibiting effects of the five fracitons on β-hexosaminidase release in RBL-2H3 cells.
(TIF)
