# Supplementary figures and images for: The Inhibition of Mast Cell Activation of Radix Paeoniae alba Extraction Identified by TCRP Based and Conventional Cell Function Assay Systems
Source: PLoS One. 2016 May 19;11(5):e0155930. doi: 10.1371/journal.pone.0155930 (PMC4873249; doi:10.1371/journal.pone.0155930)

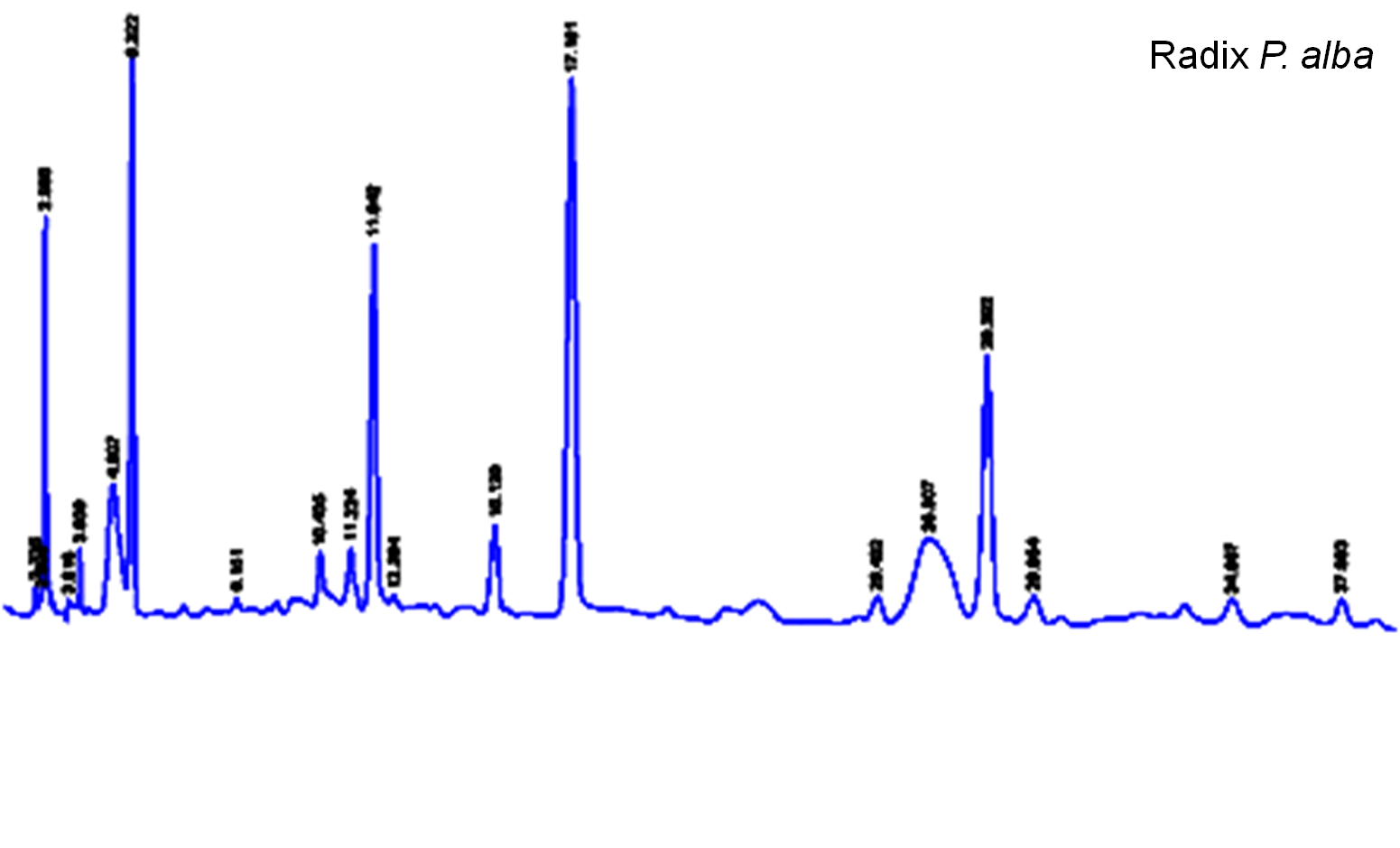

Supplement: S1 Fig — The 0.5g powered medicinal materials was added 20 mL methanol, soaked for 4h, and ultrasonic treatment for 30 min. The filtrate was 0.45 μm for filtration. Elution condition sees the Methods. (TIF) [file pone.0155930.s002.tif]

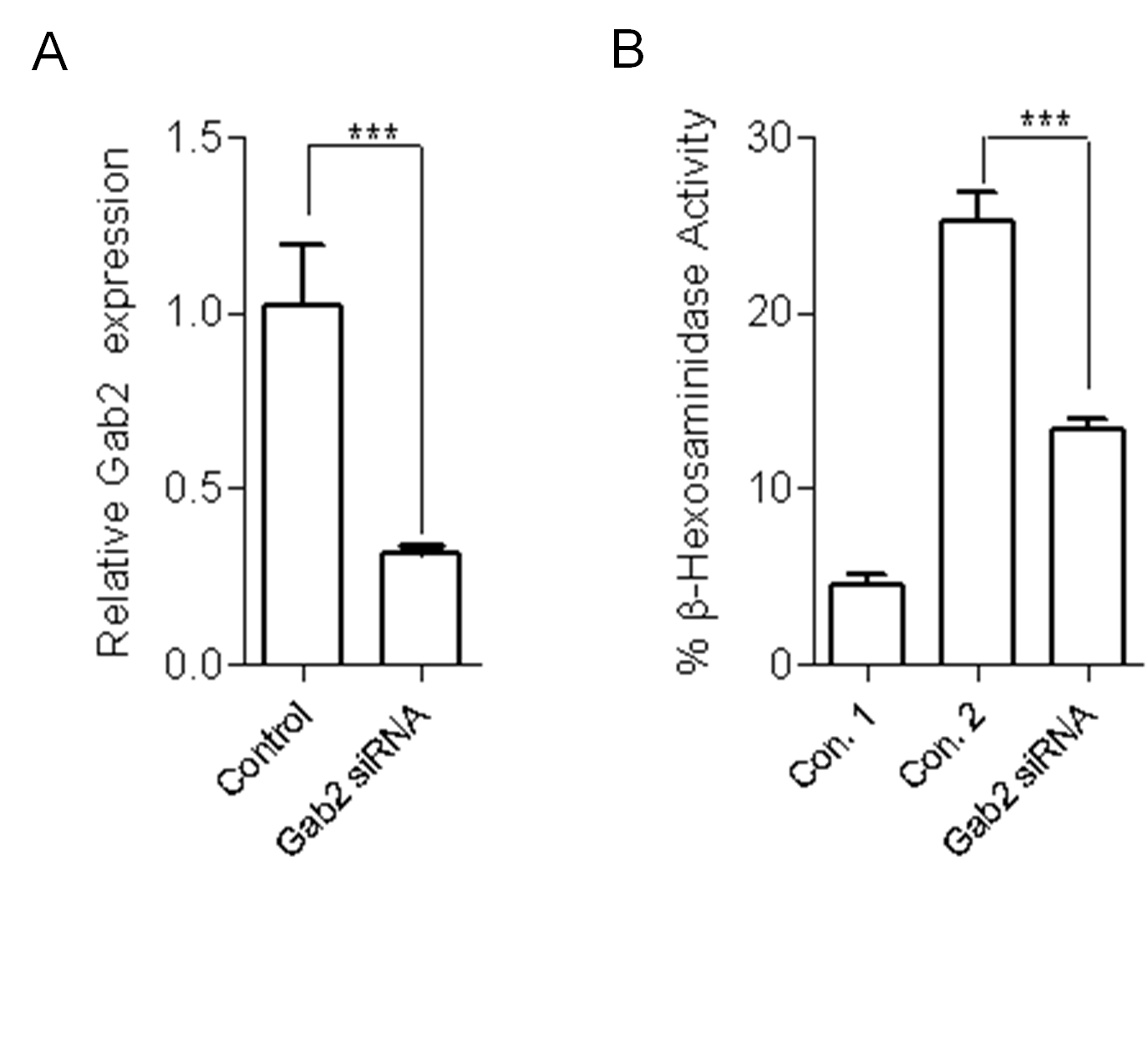

Supplement: S2 Fig — (A) Gab2 mRNA level assay of mast cells transfected with Gab2 siRNA. (B) The β-hexosaminidase release of mast cells transfected with Gab2 siRNA was decreased comparing with the only Lip 2000 treatment. The methods of β-hexosaminidase assay see the Methods. (TIF) [file pone.0155930.s003.tif]

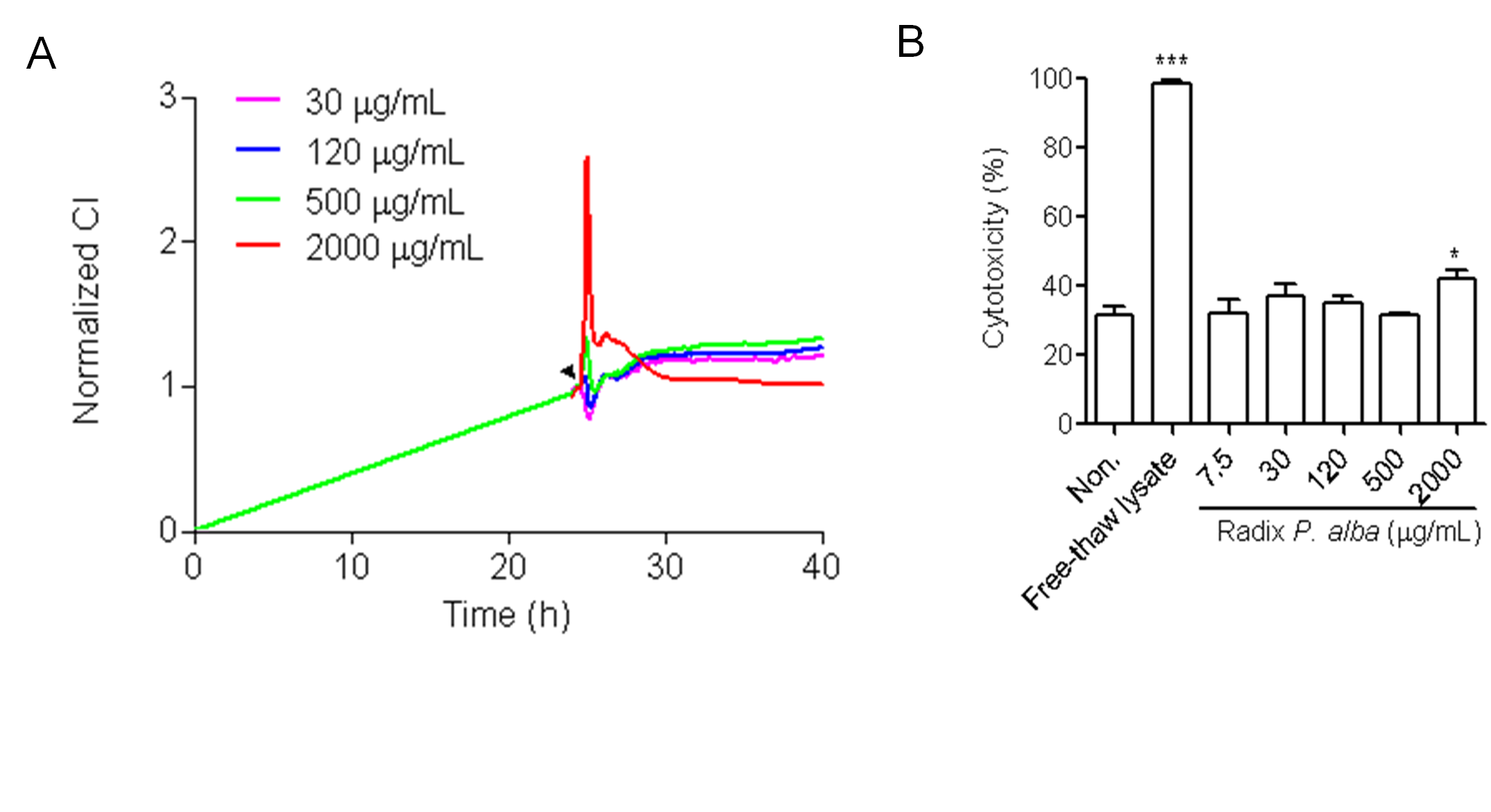

Supplement: S3 Fig — (A) The cytotoxicity observation of Radix P. alba with TCRP approach. RBL-2H3 cells were seed on the E-plate allowing to grow for 24 h, and then were added the various concentrations of Radix P. alba monitoring every 15 min for over 20 h. (B) Cytotoxicity assay of Radix P. alba on RBL-2H3 cells. RBL-2H3 cells were cultured in a 96-well plate at 10,000 cells per well for 24h and treated with various concentrations of Radix P. alba for 2h. The cell supernatant was collected and assayed the LDH release with the Cyto Tox96 Non-Radioactive Cytotoxicity assay kit following the manufacturer’s suggested protocol. (TIF) [file pone.0155930.s004.tif]

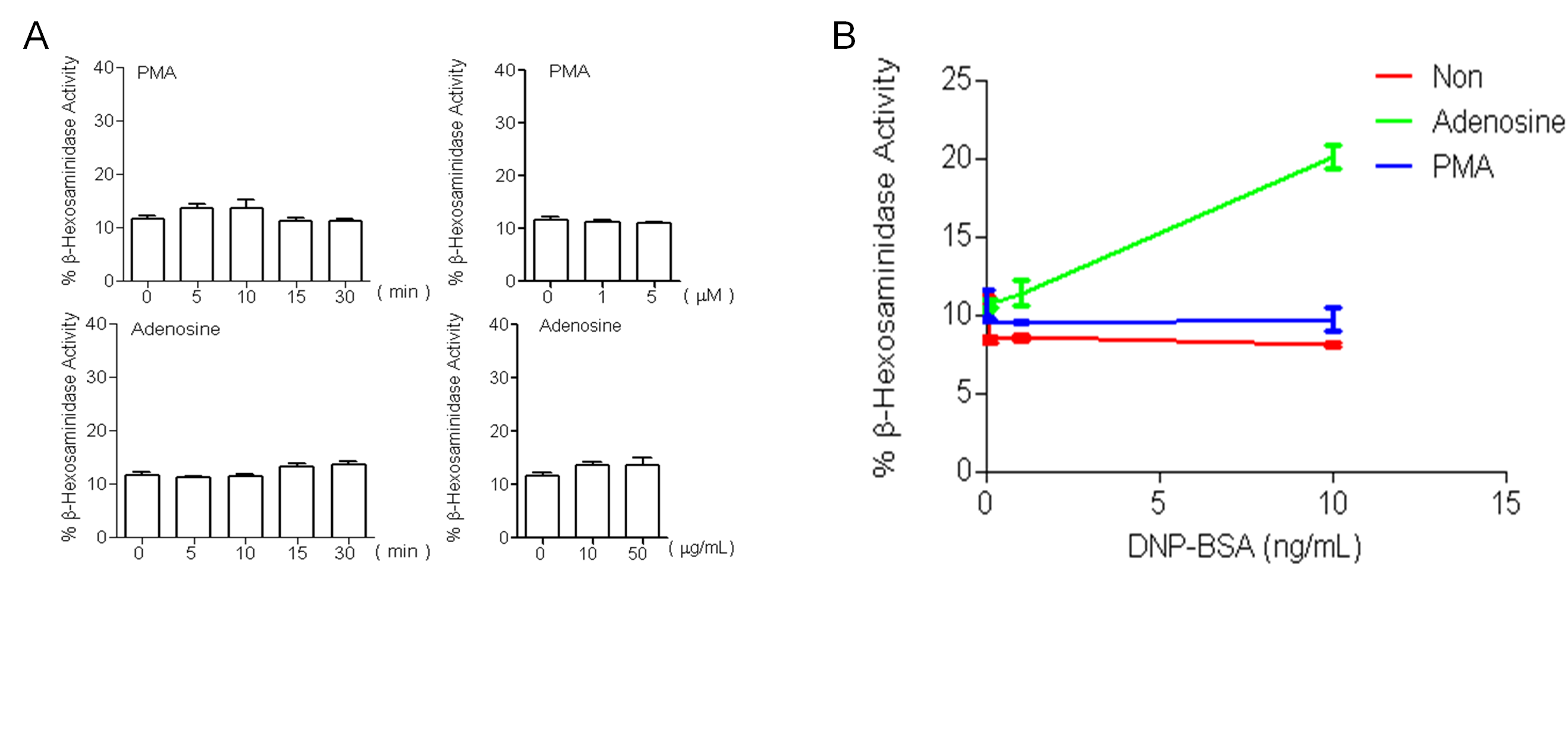

Supplement: S4 Fig — (A) The β-hexosaminidase release assays of PMA or adenosine on mast cell alone. (B) The enhancing effects of adenosine on β-hexosaminidase release from mast cells sensitized with anti-DNP IgE, when the dose of DNP-BSA fail to induce degranulation. RBL-2H3 cells were sensitized with 100 ng/mL of IgE for 24 h and stimulated with adenosine or PMA for 1min, then added the DNP-BSA with the indicated concentrations for 30min. (TIF) [file pone.0155930.s005.tif]

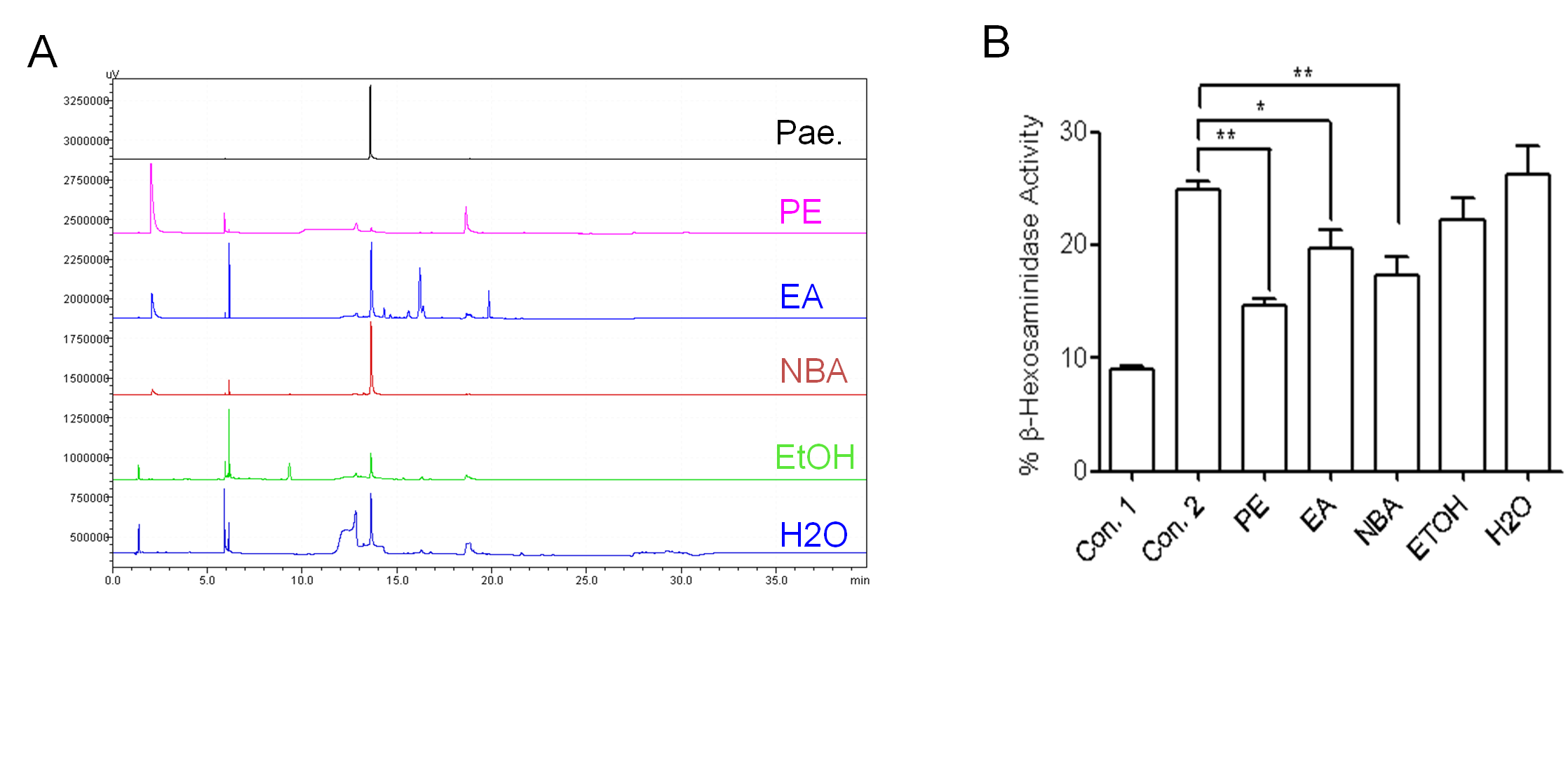

Supplement: S5 Fig — (A) The HPLC profiles of the five fractions and the control substance Paeoniflorin. (B) The inhibiting effects of the five fracitons on β-hexosaminidase release in RBL-2H3 cells. (TIF) [file pone.0155930.s006.tif]
